# Comparison of DeepSeek AI and Human Reviewers in Structured Evaluation of Neurosurgical Manuscripts: A Pilot Study

**DOI:** 10.1055/a-2964-9336

**Published:** 2026-10-09

**Authors:** Kishore Balasubramanian, Daniel O’Connor, Beste Gülsuna, Visish M. Srinivasan, Daniel A. Donoho, Nicholas Sader, Christopher S. Graffeo

**Affiliations:** 1 Department of Neurosurgery 12308 The University of Oklahoma College of MedicineOklahoma CityOklahomaUnited States; 2 Department of Neurosurgery 14640 University of Pennsylvania Perelman School of MedicinePhiladelphiaPennsylvaniaUnited States; 3 Division of Neurosurgery 549375 Children’s National Medical CenterWashingtonDistrict of ColumbiaUnited States; 4 Department of Neurosurgery 12308 University of Oklahoma Health Sciences CenterOklahoma CityOklahomaUnited States

**Keywords:** peer review, artificial intelligence, large language model, DeepSeek, neurosurgery

## Abstract

**Objectives** Increasing manuscript volume has strained peer review, prompting interest in artificial intelligence (AI) as a supportive tool. We evaluated DeepSeek R1 as a structured peer reviewer of neurosurgical case-based manuscripts and compared its scoring patterns with those of an experienced editor and student reviewers.

**Design** Exploratory, cross-sectional comparative study using a convenience sample of consecutively submitted manuscripts.

**Setting** Single skull-base neurosurgical journal during a 6-month submission window (January–June 2025).

**Participants** Three reviewer groups independently assessed 11 de-identified, unpublished neurosurgical manuscripts: first-year medical students ( *n* = 154; each randomly assigned one manuscript after a course in biostatistics, epidemiology, and critical appraisal), the journal’s Editor-in-Chief (Experienced Reviewer), and DeepSeek R1.

**Main Outcome Measures** Each manuscript was rated on a 3-point Likert scale (1 = low, 2 = intermediate, 3 = high) across five domains: Study Objective, Novelty, Study Design, Thoroughness, and Clarity.

**Results** Significant between-group differences were found in four of five domains: Study Objective ( *H* = 8.31, *p* = 0.02), Novelty ( *H* = 9.74, *p* = 0.008), Thoroughness ( *H* = 10.49, *p* = 0.005), and Clarity ( *H* = 8.13, *p* = 0.02); Study Design did not differ ( *H* = 1.45, *p* = 0.483). DeepSeek scored significantly higher than the Experienced Reviewer for Study Objective ( *p* = 0.015) and higher than both human groups for Novelty.

**Conclusions** DeepSeek showed consistency in structured domains but systematically inflated subjective ratings, particularly Novelty. A human-in-the-loop model integrating AI efficiency with expert judgment may optimize peer review.

## Introduction

Peer review is an integral component of academic scholarship and the broader enterprise of advancing scientific knowledge. It helps ensure the quality and validity of research and relies on a system of volunteerism in which investigators read and review their peers’ submitted work. However, the expanding volume of submissions has led to challenges in finding qualified reviewers, longer review times, and mounting concerns with regard to the overall fairness of the process. 1 In tandem, scholarship has become an increasingly emphasized component of undergraduate medical education. 2 Although numerous novel efforts have been developed to teach students about the principles of good study design, research methods, or critical appraisal, peer review remains essentially unmentioned during medical school. 3

Artificial intelligence (AI) has emerged as a potential avenue to address emerging challenges with peer review. 4 AI-based large language models (LLMs) have delivered promising results in accurately parsing, processing, and generating human-like text across various domains. AI-driven automated systems have been widely and successfully integrated into manuscript submission platforms, assisting in plagiarism detection, reference verification, duplicate submission checks, and compliance with journal policies. 5 These successes have prompted growing interest in deploying AI across various aspects of the peer review process, including primary assessment of submitted manuscripts. 6

DeepSeek is a family of open-weight LLMs designed for efficient reasoning and domain adaptation, with commercially permissive licensing that facilitates fine-tuning and private deployment. Although DeepSeek has demonstrated strong performance on general reasoning and technical benchmarks, there are no published data validating its use as a primary tool for scientific peer review, particularly in neurosurgery. 7 8 9 Unlike traditional AI tools, DeepSeek can be, in theory, specifically optimized for complex tasks, such as peer review of scientific manuscripts, with capabilities that should include methodological analysis, bias assessment, and novelty evaluation. 5 10 11 With the introduction of each new AI model, a step change has been observed with regard to the capabilities and potential applications across academic domains.

Recent PubMed-indexed studies confirm that AI LLMs demonstrate proficiency in structured tasks while showing a tendency to inflate scores in subjective domains such as novelty assessment in clinical peer review, raising concerns about bias in high-stakes evaluation in specialized fields like neurosurgery where case rarity may be conflated with true innovation. 12 13 No prior neurosurgery-specific AI peer-review benchmarks exist, justifying this comparison. Although we focus on neurosurgery, this concern is not confined to a single discipline: Case reports and case series remain among the most commonly published studies across surgical subspecialties and, despite their lower position in the evidence hierarchy, are among the most frequently cited article types informing point-of-care surgical practice. 14 The findings reported here may therefore have relevance for the broader landscape of case-based surgical scholarship.

In this pilot study, the focus is not on reproducing the full scope of journal peer review, but on a constrained, structured component of that process. Accordingly, the present work should be interpreted as a preliminary comparison of domain-specific rating patterns among an experienced editor, novice reviewers, and the DeepSeek AI model in this specific structured rating task, rather than a comprehensive evaluation of DeepSeek as a standalone peer reviewer.

## Methods

### Study Design

This comparative analysis evaluated DeepSeek’s performance as an AI peer reviewer against human counterparts. This study was exempt from Institutional Review Board review. Eleven unpublished observational studies on neurosurgical topics ( Table 1 ), submitted to the *Journal of Neurological Surgery Reports* , were selected consecutively from submissions between January and June 2025. Selection criteria included (1) primary neurosurgical focus (e.g., cranial, spinal, or endovascular cases); (2) observational design (case reports/series); (3) completeness of submission package (manuscript, figures, tables); excluding those with ethical conflicts or incomplete data. To minimize selection bias, consecutive submissions meeting these criteria during the study period were used, without preselection for topic, perceived quality, or novelty.

**Table 1:** Study summaries.

Study number	Case report title	Brief description
1	Chronic-subacute subdural hematoma mimicking meningioma	First reported case of organized hematoma mimicking convexity meningioma on contrast-enhanced MRI
2	Bilateral endoscopic optic nerve decompression in infant with osteopetrosis	First pediatric osteopetrosis case using cost-effective endoscopic decompression pre-BMT with 6-week follow-up
3	Complete hearing recovery after jugular foramen schwannoma resection	**First documented hearing restoration in a patient with concurrent vestibular and jugular foramen schwannoma using a tailored retrosigmoid approach**
4	Keyhole mini-craniotomy for tegmen repair	Validates minimally invasive approach for middle fossa defects with reduced hospitalization versus traditional methods
5	VP shunt protruding through anus	Reports conservative management of spontaneous anal shunt extrusion in resource-limited setting
6	Pulsating oral cavity mass from carotid aneurysm	Presents third reported case of carotid aneurysm presenting as oral pulsatile mass with SSCD surgical adaptation
7	Complete resorption of cranial bone flap	Rare bone flap resorption with successful PEEK implant reconstruction and inflammatory mechanism analysis
8	Retropharyngeal schwannoma via Smith Robinson approach	Demonstrates successful schwannoma resection in NF2 patient using anterior cervical approach with imaging correlation
9	Giant lateral ventricular meningiomas in low-income country	Ten-year surgical outcomes ( *n* = 9) using transcortical-parietal approach without neuronavigation in Peru
10	HDAC2 inhibitors in chronic pancreatitis pain	Mechanistic study showing ERK-Sp1 pathway regulates HDAC2 in spinal pain sensitization using rat CP model
11	K _i_ -67 and OCT correlation in pituitary tumors	Links socioeconomic factors (CDC SVI) to OCT imaging biomarkers in pituitary adenomas ( *n* = 34)

SSCD: Superior semicircular canaldehiscence syndrome; PEEK: This is a proper noun; OCT: optical coherence tomography; CDC:Centers for Disease Control; SVI: social vulnerability index; CP: Chronic pancreatitis; ERK-Sp1: This is a proper noun; NF2: This is a proper noun; HDAC2: This is a proper noun

All 11 manuscripts were active or recently completed submissions to *Journal of Neurological Surgery Reports* and were accessed by the journal’s Editor-in-Chief (EIC) in his routine editorial capacity. For this study, manuscripts were de-identified before any student or AI involvement, with author names, affiliations, and other direct identifiers removed. No manuscript texts were shared outside the study team and student participants; students accessed only a single de-identified manuscript for educational and research purposes under faculty supervision. DeepSeek evaluations were generated by uploading de-identified manuscripts to the DeepSeek platform using a study-specific account, and no task-specific fine-tuning or additional training on neurosurgical content was performed.

This study was designed as an exploratory, cross-sectional comparison of structured domain scores across three reviewer types, using a convenience sample of manuscripts and a single expert reference standard; it was not powered or intended to establish formal non-inferiority or equivalence between AI and human peer review.

### Reviewer Groups

These studies were independently assessed by three distinct reviewer groups: first-year medical students who had recently completed a course on critical appraisal ( *n* = 154, each randomly assigned 1 of the 11 studies), the journal’s EIC (serving as the Experienced Reviewer), and DeepSeek’s AI system. The Experienced Reviewer was the EIC of *Journal of Neurological Surgery Reports* , qualified by 3 years in that position with at least 10 preceding years of editorial and peer-review experience encompassing >250 manuscript reviews, in addition to personal authorship of >300 PubMed-indexed publications (H-index 34). Although the medical students had no prior experience serving as peer reviewers, they represent a population increasingly exposed to research training. Their evaluations in this study are therefore best interpreted as structured ratings from trained novices, not as surrogates for expert peer review. First-year medical students were selected deliberately as a structured-but-inexperienced human comparator whose limited domain expertise approximates that of a general-purpose AI model applying explicit criteria without accumulated editorial experience. This design allowed the AI system to be positioned along a spectrum bounded by an expert editor at one end and trained novices at the other, isolating the effect of experience on rating behavior. More experienced comparators, such as residents or early-career attendings, would provide a stronger benchmark for editorial judgment but would also introduce heterogeneous prior review experience and reduced availability; their inclusion is an important direction for future work.

### Review Process

Each submission underwent evaluation across five critical domains using the same 3-point Likert scale for each (1 = low quality, 2 = intermediate quality, 3 = high quality): Study Objective (clarity of research aims), Novelty (contribution to existing knowledge), Study Design (methodological rigor), Thoroughness (completeness of investigation), Clarity (clarity of presentation). Among the five domains, Study Objective, Study Design, and Clarity emphasize relatively more structured, “objective-learning” features (e.g., presence of a clear research question, consistency of methods, adherence to conventional reporting standards), whereas Novelty and Thoroughness require more interpretive judgment of incremental contribution and perceived completeness. The domains were selected a priori to capture core elements of clinical case report quality while remaining feasible for novice raters and an AI model.

For each manuscript, domain scores were summed to generate a total score ranging from 5 to 15, which served as the primary comparative outcome across student, experienced editor, and DeepSeek reviewers. The scorecard was not formally validated as a measure of manuscript quality and should be interpreted as a standardized framework for comparing rating patterns across reviewer groups rather than as a definitive quality assessment tool.

All AI evaluations were generated using the DeepSeek R1 model accessed via the official DeepSeek platform. For this study, default parameters were used, and no fine-tuning or additional training on neurosurgical content was performed. DeepSeek’s internal training data and optimization procedures are proprietary and not publicly disclosed; as such, the present work should be understood as a black-box evaluation of model behavior under specified prompting conditions.

Each manuscript was uploaded as a complete portable document format (PDF) or text file, followed by a standardized prompt instructing the model to read the full article and produce ratings for the five domains. The prompt text matched, verbatim, the written instructions provided to human reviewers ( **Supplemental Fig. S1** [available in the online version only]). AI outputs consisted of discrete numerical scores; free-text explanations, when generated, were not analyzed in this study. The Experienced Reviewer was blinded to other scores; student scores ( *n* = 154, randomly assigned per study post-biostatistics/epidemiology course) were averaged per study.

Continuous variables were reported as medians with interquartile ranges (IQRs). Given the small number of manuscripts and the ordinal nature of the ratings, the Kruskal–Wallis tests and post hoc pairwise comparisons (Dwass–Steel–Critchlow–Fligner) were used to compare score distributions across groups; no claims are made regarding the absolute validity of any group’s ratings, which were not benchmarked against a consensus panel or external gold standard. Jamovi (The Jamovi Project, open source) was utilized for all statistical analyses.

## Results

Using this five-domain scorecard, the performances of DeepSeek, first-year medical students, and the Experienced Reviewer were evaluated across 11 clinical studies using a scorecard with five domains: Study Objective, Novelty, Study Design, Thoroughness, and Clarity ( Fig. 1 and Table 2 ).

**Fig. 1:**
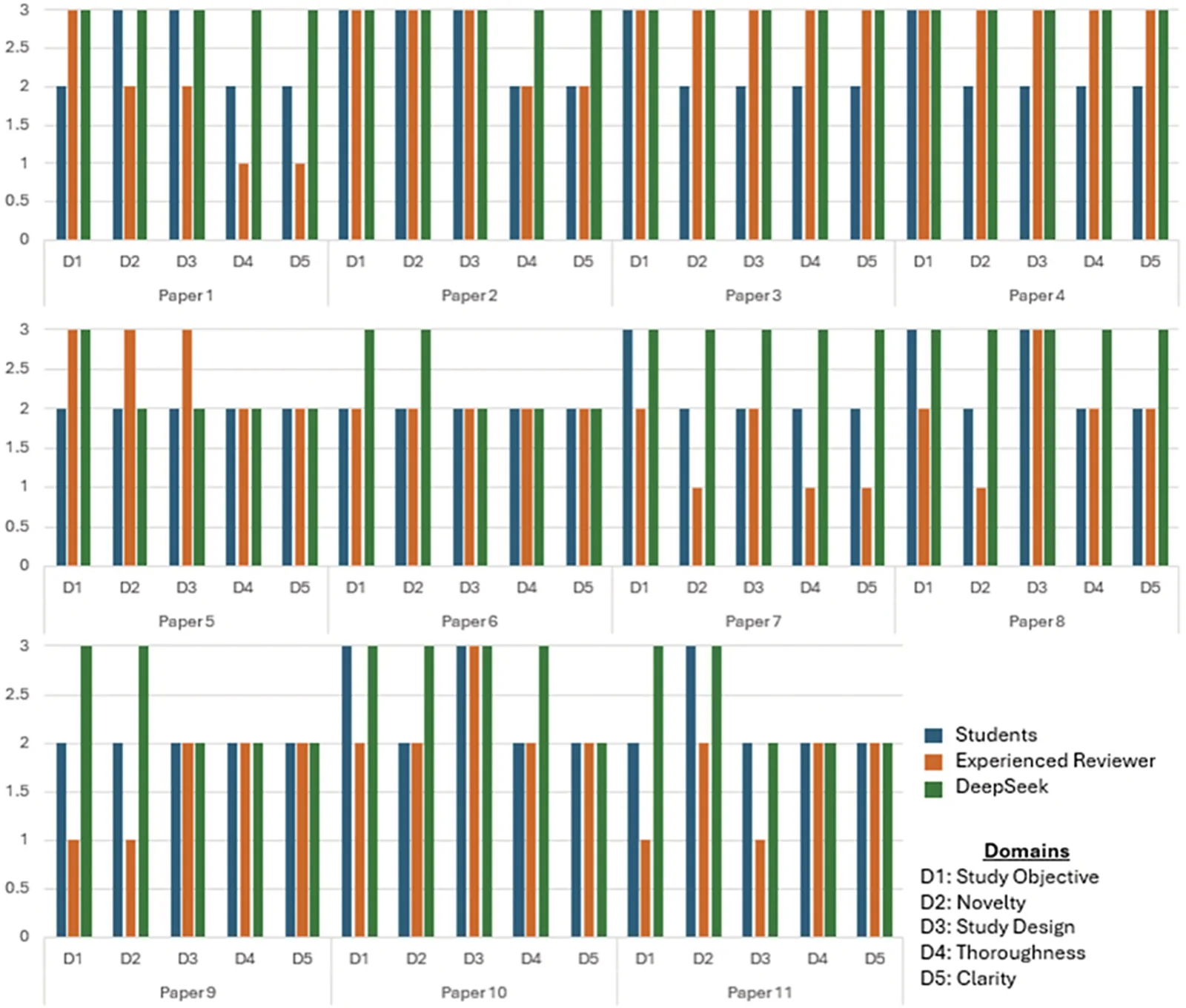
Performance by study and domain.

**Table 2:** Summary data.

Medical students												
	Paper 1	Paper 2	Paper 3	Paper 4	Paper 5	Paper 6	Paper 7	Paper 8	Paper 9	Paper 10	Paper 11	Total
Study Objective	2	3	3	3	2	2	3	3	2	3	2	28
Novelty	3	3	2	2	2	2	2	2	2	2	3	25
Study Design	3	3	2	2	2	2	2	3	2	3	2	26
Thoroughness	2	2	2	2	2	2	2	2	2	2	2	22
Clarity	2	2	2	2	2	2	2	2	2	2	2	22
Total	12	13	11	11	10	10	11	12	10	12	11	
**Experienced Reviewer**												
	**Paper 1**	**Paper 2**	**Paper 3**	**Paper 4**	**Paper 5**	**Paper 6**	**Paper 7**	**Paper 8**	**Paper 9**	**Paper 10**	**Paper 11**	**Total**
Study Objective	3	3	3	3	3	2	2	2	1	2	1	25
Novelty	2	3	3	3	3	2	1	1	1	2	2	23
Study Design	2	3	3	3	3	2	2	3	2	3	1	27
Thoroughness	1	2	3	3	2	2	1	2	2	2	2	22
Clarity	1	2	3	3	2	2	1	2	2	2	2	22
Total	9	13	15	15	13	10	7	10	8	11	8	
**DeepSeek**												
	**Paper 1**	**Paper 2**	**Paper 3**	**Paper 4**	**Paper 5**	**Paper 6**	**Paper 7**	**Paper 8**	**Paper 9**	**Paper 10**	**Paper 11**	**Total**
Study Objective	3	3	3	3	3	3	3	3	3	3	3	33
Novelty	3	3	3	3	2	3	3	3	3	3	3	32
Study Design	3	3	3	3	2	2	3	3	2	3	2	29
Thoroughness	3	3	3	3	2	2	3	3	2	3	2	29
Clarity	3	3	3	3	2	2	3	3	2	2	2	28
Total	15	15	15	15	11	12	15	15	12	14	12	

Medical students’ cumulative scores exhibited a narrow range from 10 to 13 (median = 11, IQR = 2), with most domain-specific ratings clustering at the intermediate value of 2, consistent with central tendency bias commonly seen in inexperienced raters ( Fig. 2 ). In contrast, the Experienced Reviewer assigned the full range of available scores (7–15, median 10), while DeepSeek’s ratings were skewed toward the upper end of the scale (11–15, median 15), indicating a calibration toward higher ratings relative to the Experienced Reviewer and students, rather than demonstrably higher manuscript quality. A comparable trend was also seen when scores were evaluated by domain ( Fig. 3 ).

**Fig. 2:**
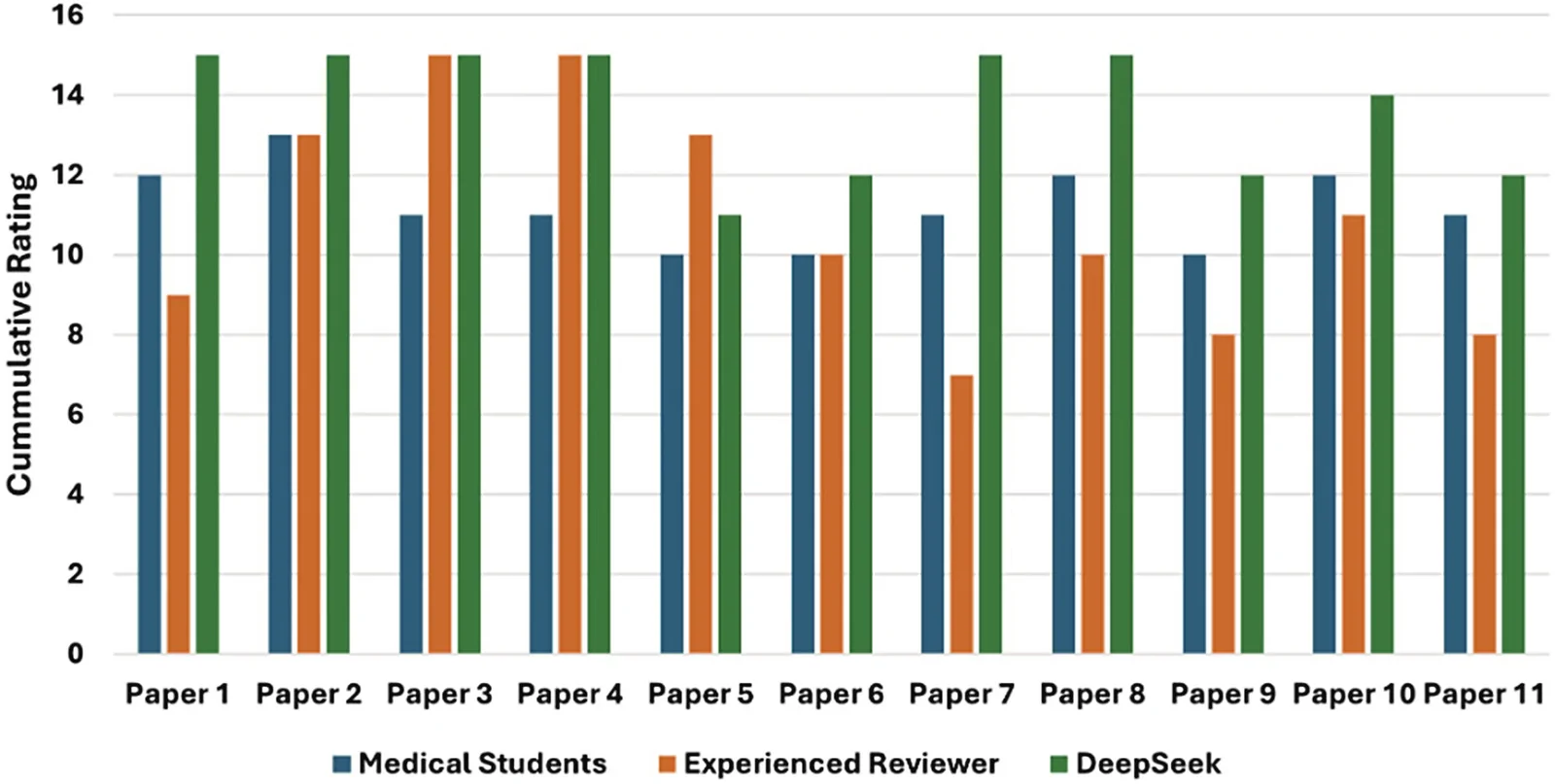
Performance by study.

**Fig. 3:**
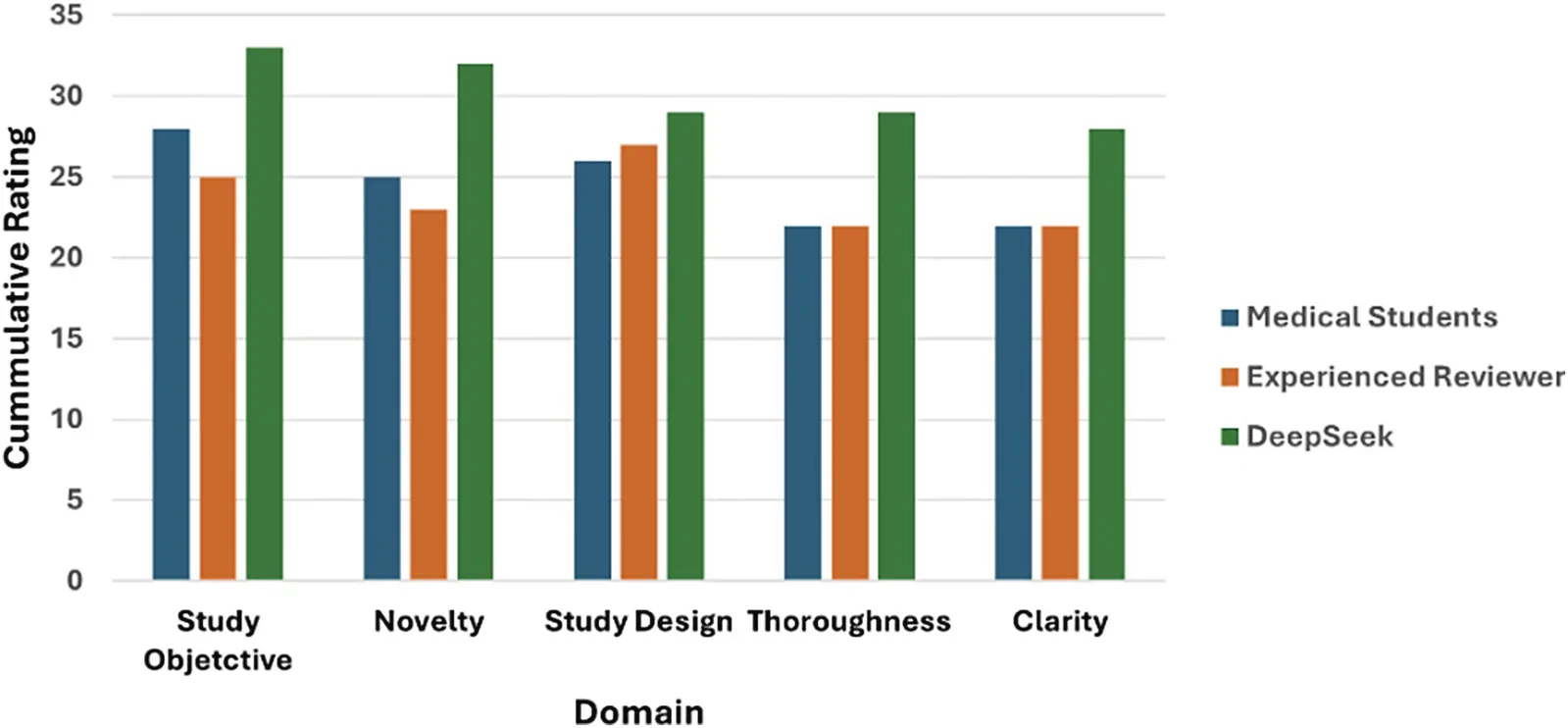
Performance by domain.

These differences were most pronounced in the Study Objective and Novelty domains, where DeepSeek’s ratings were significantly higher. When evaluating the overall distribution of ratings given by each group ( Fig. 4 ), the experienced reviewer provided a near-normal distribution of low, intermediate, and high ratings. The medical students, on average, reported a left-skewed ratings distribution, while the ratings distribution reported by DeepSeek was right-skewed. These trends were also seen when evaluating the score distribution by domain ( **Supplemental Fig. S2** [available in the online version only]).

**Fig. 4:**
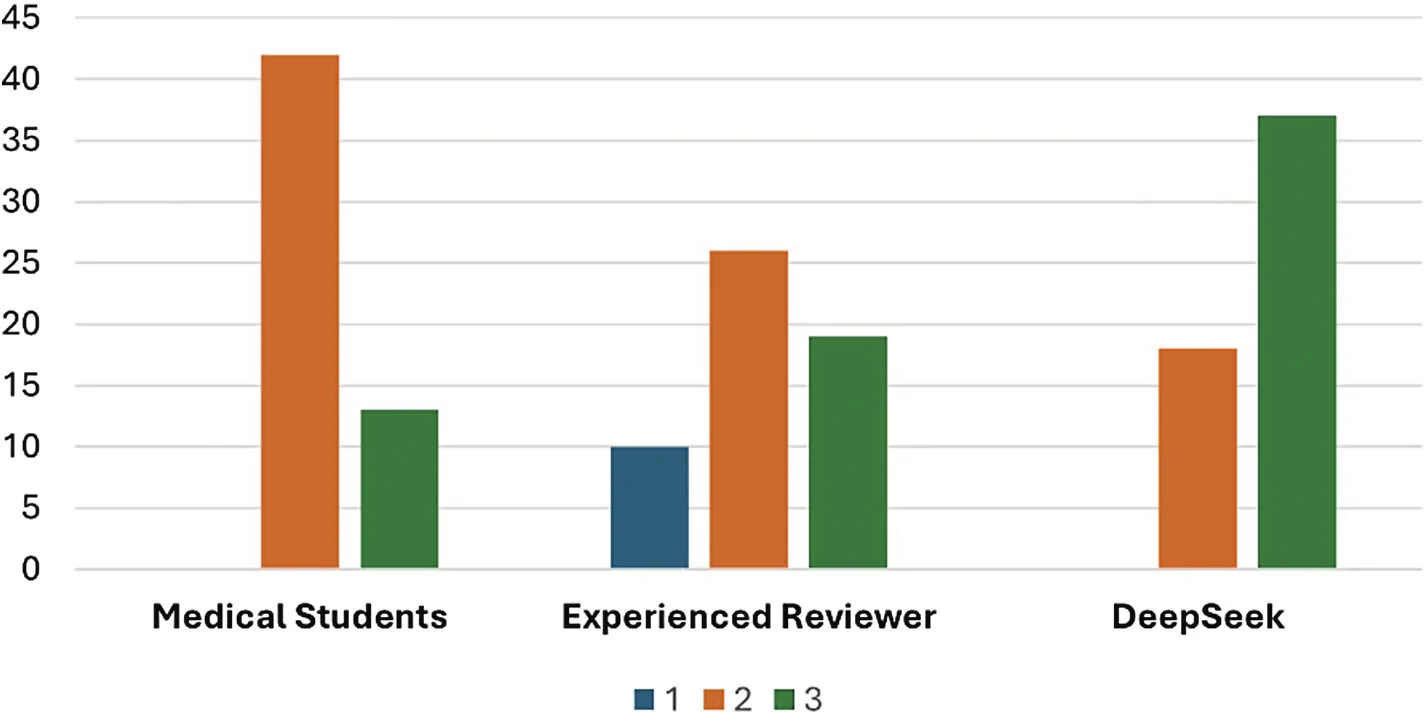
Distribution of overall ratings.

Significant differences between the three reviewer groups were observed in four of the five study domains (Study Objective *H* = 8.31, *p* = 0.02; Novelty *H* = 9.74, *p* = 0.008; Thoroughness *H* = 10.49, *p* = 0.005; Clarity *H* = 8.13, *p* = 0.02; Table 3 ), while Study Design showed no significant differences ( *H* = 1.45, *p* = 0.483).

**Table 3:** Differences among groups by domain.

Kruskal–Wallis		
Domain		Kruskal–Wallis *H* -value, *p* -value
Study Objective		*H* = 8.31, *p* = 0.02 ^a^
Novelty		*H* = 9.74, *p* = 0.008 ^a^
Study Design		*H* = 1.45, *p* = 0.483
Thoroughness		*H* = 10.49, *p* = 0.005 ^a^
Clarity		*H* = 8.13, *p* = 0.02 ^a^
**Post hoc test**		
**Domain**	**Comparison**	** Dwass–Steel–Critchlow–Fligner *W* -value, *p* -value **
Study Objective	DeepSeek versus Experienced Reviewer	*W* = 3.92, *p* = 0.015
Novelty	DeepSeek versus Experienced Reviewer	*W* = 3.75, *p* = 0.022
Novelty	DeepSeek versus Medical Students	*W* = 4.19, *p* = 0.008
Thoroughness	DeepSeek versus Medical Students	*W* = 4.43, *p* = 0.005
Clarity	DeepSeek versus Medical Students	*W* = 3.97, *p* = 0.014

Post hoc testing indicated that DeepSeek ratings were significantly higher than those of the Experienced Reviewer in the Study Objective domain ( *W* = 3.92, *p* = 0.02); significantly higher than both the Experienced Reviewer ( *W* = 3.75, *p* = 0.02) and the medical student reviewers ( *W* = 4.19, *p* = 0.008) in the Novelty domain; and significantly higher than the students but not the Experienced Reviewer in the Thoroughness ( *W* = 4.19, *p* = 0.014) and Clarity ( *W* = 4.19, *p* = 0.017) domains.

When analyzing individual studies, significant differences among the three groups were found in 5 of the 11 studies ( Table 4 ). Post hoc tests for these studies revealed that DeepSeek’s ratings were significantly higher than the Experienced Reviewers’ in three of the five studies and higher than medical students’ ratings in two of the five studies.

**Table 4:** Differences among groups by study.

Study	Kruskal–Wallis *H* -value, *p* -value	
1	*H* = 6.78, *p* = 0.034	
2	*H* = 2.55, *p* = 0.28	
3	*H* = 10.18, *p* = 0.006	
4	*H* = 10.18, *p* = 0.006	
5	*H* = 4.45, *p* = 0.108	
6	*H* = 4.31, *p* = 0.116	
7	*H* = 10.94, *p* = 0.004	
8	*H* = 6.53, *p* = 0.038	
9	*H* = 5.60, *p* = 0.061	
10	*H* = 3.50, *p* = 0.174	
11	*H* = 4.38, *p* = 0.087	
**Post hoc test**		
**Study**	**Comparison**	** Dwass–Steel–Critchlow–Fligner *W* -value, *p* -value **
1	Experienced Reviewer versus DeepSeek	*W* = 3.35, *p* = 0.047 ^a^
3	Medical Students versus Experienced Reviewer	*W* = 3.46, *p* = 0.038 ^a^
3	Medical Students vs DeepSeek	*W* = 3.46, *p* = 0.038 ^a^
4	Medical Students versus Experienced Reviewer	*W* = 3.46, *p* = 0.038 ^a^
4	Medical Students versus DeepSeek	*W* = 3.46, *p* = 0.038 ^a^
7	Experienced Reviewer versus DeepSeek	*W* = 4.01, *p* = 0.013 ^a^
8	Experienced Reviewer versus DeepSeek	*W* = 3.38, *p* = 0.044v

## Discussion

We report, to our knowledge, the first study in the neurosurgical literature examining DeepSeek’s behavior in a structured rating task applied to neurosurgical manuscripts, alongside an experienced editor and novice reviewers. The present study does not establish DeepSeek as a valid or sufficient tool for scientific evaluation; rather, it describes how one AI model scores a small set of neurosurgical manuscripts relative to a single expert and a cohort of novices.

### Study Design Considerations

The choice to use a single experienced editor as the expert reference standard reflects common editorial practice, in which editors frequently make initial decisions or final judgments based on their own assessment and selected peer reviews. However, empirical studies consistently demonstrate that inter-reviewer agreement in grant and manuscript peer review is modest, particularly for subjective dimensions such as significance or innovation. 15 16 17 As a result, the present comparison to a single expert should be interpreted as an evaluation of concordance with one established editorial viewpoint, not as an estimation of consensus expert judgment.

Similarly, the inclusion of first-year medical students provides insight into how trained novices apply structured criteria but does not constitute a benchmark for expert peer review. Their marked central tendency pattern and limited experience suggest that their ratings are best understood as a distinct behavioral profile, rather than as a ground truth for manuscript quality or AI performance.

Thus, the comparator groups (a single Experienced Reviewer, a large cohort of novice student reviewers, and a single AI model) were selected to represent a spectrum from expert editorial judgment to early training perspectives and algorithmic behavior, but do not provide a formal reference standard for “correct” peer review decisions. Future work should incorporate multiple expert reviewers, consensus-based reference ratings, and additional AI comparators to more robustly evaluate reliability, validity, and generalizability of AI-assisted peer review.

### Rating Calibration and Score Distributions

DeepSeek demonstrated systematic rating disparities as compared with human evaluators, assigning maximum scores (15/15) to 45% of papers, a score that was noted in 0% of the medical students’ reviews and only 18% of Experienced Reviewer ratings. This gradient in score distributions mirrors prior observations of score inflation by algorithmic systems in scientific evaluation tasks. 4 While the magnitude of disparity varied significantly by domain, the observation of the Novelty domain showing the largest gap (DeepSeek = 32/33 vs. expert = 23/33) strengthens the view of AI leniency. Given the lack of training data on this specific task and thus the inability to develop a clinical context, AI models seem to interpret incremental clinical observations as novel contributions, or at the very least, they take authors at their word. This is in stark contrast to human reviewers, who bring both expertise and skepticism to this determination. This observation aligns with preceding studies demonstrating the broad failure of AI models such as LLMs to accurately assess innovation. 4 18

### Differences in Rating Behavior

The Experienced Reviewer showed a greater range of scores as compared with DeepSeek (7–15 vs. 11–15) and assigned the only lowest-possible scores across all ratings, reflecting the experience and nuanced, critical perspective that reflect both clinical experience and comfort with critical appraisal. This finding mirrors Mostafapour et al, who noted that expert reviewers employ stricter clinical relevance filters as compared with AI models. 12 Conversely, medical students displayed conservatism, with most ratings clustered around the intermediate score of 2, likely reflecting their self-consciousness and lack of experience as novice reviewers. Notably, this conservatism was asymmetric: students withheld the lowest available rating entirely, yet were willing to assign the highest rating on occasion. This pattern is consistent with a reluctance to appear overly critical, which is plausibly heightened by their inexperience and by the evaluative context, coupled with a comparatively lower threshold for endorsing perceived strengths. A tendency to avoid the harshest judgment, rather than to avoid extreme judgments altogether, may thus better characterize novice rating behavior in this setting than symmetric central tendency bias alone. DeepSeek, by contrast, was heavily biased toward more favorable scores, a finding that aligns with previous observations demonstrating how the lack of contextual richness of AI models results in an overvaluing of incremental findings as being highly novel or impactful. 12

### Domain Specific Performance

Study Design evaluations showed remarkable interrater concordance (Kruskal–Wallis *p* = 0.483), suggesting that structured reporting elements enable reliable assessment across experience levels and are complementary with the reasoning strategies implemented by AI models. 19 Conversely, Novelty assessments produced significant discordance ( *p* = 0.008), with DeepSeek disproportionately rewarding conventional clinical research (Papers 5, 6, and 9 scored 2–3 by humans vs. AI’s 3). Taken together, this indicates that, although contemporary AI systems are adequately equipped for tasks with structured elements and outcomes, their lack of contextual frameworks may impede accurate or useful discernment of genuine innovation and novelty.

### Human-in-the-Loop Model

DeepSeek’s consistency in Study Design scoring suggests that AI may be effectively applied to standardized tasks such as assessing manuscript formatting, alignment with journal scope, and appropriateness of statistical methods, potentially reducing key aspects of the administrative burden associated with peer review. However, its limited discriminatory capacity in nuanced domains such as novelty assessment highlights a core risk of fully autonomous AI-based peer-review workflows. These findings align with the broader framework of human-in-the-loop (HITL) AI systems, which integrate human expertise with AI efficiency. Our data offer direct empirical support for such a model. The specific pattern we observed, high interrater concordance in the structured Study Design domain alongside systematic overinflation in the interpretive Novelty domain, maps cleanly onto the division of labor that an HITL framework prescribes tasks in which the AI is reliable can be safely delegated, whereas the domains in which it is demonstrably overgenerous are precisely those that should be routed to expert human judgment. In other words, the same results that caution against autonomous AI review constitute the strongest argument for retaining a human in the loop, with the AI serving as a structured first pass rather than a final arbiter of novelty or impact.

An HITL system is an AI model that incorporates human oversight at key decision points to improve accuracy, reliability, and contextual understanding. Rather than operating independently, AI systems in an HITL model generate preliminary assessments, which are then refined by human experts who provide critical judgment, domain-specific knowledge, ethical perspective, or other inputs that are abstract or otherwise challenging to model. HITL has been successfully implemented in various fields ranging from health care diagnostics to financial auditing or automated content moderation, where AI can efficiently process large datasets, flagging critical checkpoints for human input on difficult, atypical, nuanced, or instrumental decisions.

Applying this model to peer review, one imagines an HITL framework in which AI conducts initial screenings and structured assessments (e.g., such as plagiarism detection, statistical validation, compliance checks), after which a human reviewer performs a more focused review concentrating on those areas mandating deep expertise—scientific integrity, novelty, or impact, for example. Over time, these human inputs may provide substrate for further AI model training, providing the absent contextual framework that hampers contemporary assessments, and potentially informing preliminary recommendations. Although further testing would be required, successful experience in parallel domains indicates that, as the AI models gain experience, their recommendations may become increasingly accurate, ideally yielding exponential improvements in efficient and effective peer review. 20 Conversely, AI-assisted peer reviews have been shown to impact the probability of acceptance, potentially compromising the rigorous standards that have long been a hallmark of academic publishing. 18 Notwithstanding, although the current study data provide compelling early findings that AI models will powerfully augment scholarly publishing, they also suggest that human stewardship will remain central in the contextualization and interpretation of scientific achievements. 11 21 The ideal role of AI in the peer review process is one that streamlines workflow without impacting critical review and editorial decision-making.

### Future Perspectives

Some key domains require further evaluation to optimize the integration of AI in peer review. Regarding the AI–reviewer relationship, studies should explore the extent to which AI-generated assessments influence human reviewers’ final decisions. 22 23 While AI systems provide structured feedback, it remains unclear whether these recommendations shape reviewers’ judgments or introduce cognitive biases, as well as overreliance on AI-generated suggestions. 11 20 24 Indeed, studies have shown that manuscripts that underwent AI-assisted review scored higher than those under human review. 17 Similarly, submissions near the acceptance threshold that received an AI-assisted peer review were more likely to be accepted. 17 Future studies should evaluate how AI-driven evaluations align with reviewers’ prior experiences and potential biases, ensuring that AI does not unintentionally reinforce existing disparities or hinder objectivity. 10 11 21

From a systems perspective, the optimal composition of HITL models is yet to be established. Although these appear poised to improve scientific rigor and publication efficiency by combining an AI model’s ability to process large volumes of data with a human reviewer’s expertise, context, and judgment, directed investigation is required to determine the optimal balance between human oversight and AI-driven automation. 20 25 Additionally, while AI appears highly likely to streamline repetitive tasks like plagiarism checks and statistical validation, objective studies should explicitly examine whether these time-saving measures lead to faster peer review and higher impact publications for the journal without sacrificing review quality or author satisfaction. 10 11 26 Academic journals are well-positioned to investigate this role further. Internal randomized trials evaluating features such as AI-assisted review or even AI reviewer agents can help delineate the role AI can play in an otherwise human peer-supervised process. Finally, although this pilot was conducted in neurosurgery, its implications are unlikely to be confined to a single discipline. A structured, human-supervised role for AI in triaging such case-based submissions may therefore carry relevance well beyond neurosurgery, and the domain-specific rating behavior observed here provides an early framework for evaluating that role in other surgical fields.

### Limitations

The current study is subject to several key limitations. First, it includes only 11 observational neurosurgical manuscripts from a single journal, which restricts statistical power and generalizability. Second, ratings were compared with those of a single experienced editor. As a result, concordance with the Experienced Reviewer should be interpreted as alignment with one editorial perspective rather than agreement with a broader expert benchmark.

Third, the 3-point Likert scale used in this study has not been formally validated for assessing manuscript quality and may inadequately capture nuanced judgments, especially for complex constructs such as Novelty. Fourth, DeepSeek was evaluated as a black-box system: although model documentation describes an open-weight, high-capacity LLM, the training data and optimization procedures are not publicly transparent, and no task-specific fine-tuning was performed for peer review. Our findings also pertain exclusively to DeepSeek R1, evaluated with a single standardized prompt for ordinal domain ratings; results may differ for other AI architectures, deployment settings, or more complex review tasks. Additionally, there is a possibility that with more structured prompts, the AI models may have been able to achieve greater performance. However, we felt that this might introduce a source of systematic error, as AI-optimized instructions will differ from the instructions human reviewers receive. Thus, we opted to standardize the instructions given to all groups. Finally, the study examines relative score distributions rather than downstream editorial decisions or citation outcomes, and therefore cannot assess whether AI- or human-assigned ratings are more predictive of long-term scientific impact.

## Conclusion

We report a novel study that comprehensively evaluates the AI model DeepSeek as a peer reviewer for neurosurgical clinical studies. DeepSeek demonstrated strengths in consistency and efficiency, particularly in structured domains such as Study Design. However, DeepSeek also demonstrated statistically significant shifts toward higher ratings across several domains, especially Novelty, compared with both human reviewer groups.

Taken together, the findings of the current study highlight areas where AI is equipped to streamline numerous aspects of the peer-review process and reduce administrative burdens, while ultimately reinforcing the fundamental necessity of human reviewers to exercise judgment, critical appraisal, and nuanced interpretation of potentially novel scientific findings. Accordingly, these findings should be interpreted as preliminary and hypothesis-generating, requiring validation in larger, multijournal cohorts with multiple expert reviewers and diverse AI systems before any changes to editorial practice are considered.

## Data Availability

The datasets generated or analyze d during this study are available from the corresponding author on reasonable request.

## Declaration of GenAI Use

The authors declare the use of generative AI (GAI) in the research and writing process. According to the GAIDeT taxonomy (2025), the following tasks were delegated to GAI tools under full human supervision as part of the research methodology: preliminary hypothesis testing, experimental task completion, and data collection. The GAI tool used was: DeepSeek R1. Responsibility for the final manuscript lies entirely with the authors. GAI tools are not listed as authors and do not bear responsibility for the final outcomes. Declaration submitted by: Collective responsibility.

## Supplementary Material

Ergänzendes Material Supplementary Material
