# Supplementary material for: Comparison of DeepSeek AI and Human Reviewers in Structured Evaluation of Neurosurgical Manuscripts: A Pilot Study
Source: J Neurol Surg Rep. 2026 Oct 9;87(4):e192–201. doi: 10.1055/a-2964-9336 (PMC13652684; doi:10.1055/a-2964-9336)
Supplement: Supplementary file 1 [file 10-1055-a-2964-9336_29701482.pdf]

You will be acting as a scientific reviewer for a journal. Please score the submission on the following criteria (1-3 points each, 3 being highest quality):

1. Is the clinical question or study objective clear? (1-3)
2. Is the novelty of the clinical question or study objective explained or justified? (1-3)
3. Is the study design appropriate to the question/objective? (1-3)
4. Does the study provide a thorough and accurate answer to the question/objective? (1-3)
5. Do the authors present the study results in a reasonable and comprehensible fashion? (1-3)

Supplemental Image 1: The instructions given to the AI models and the human reviewers were matched verbatim.

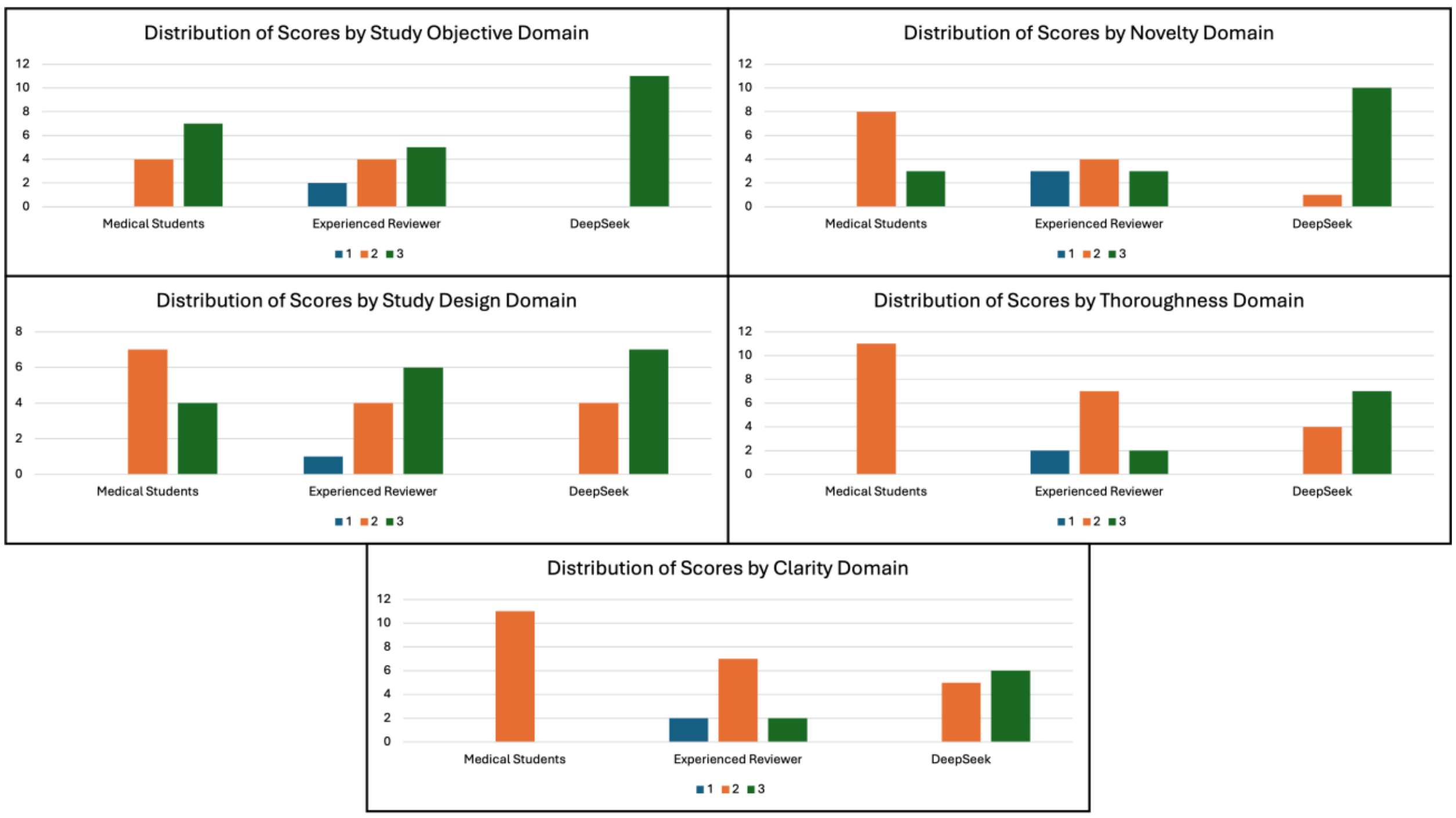

Supplemental_2_DACR: Distribution of scores by domains.
